# May the Force and Mass Be With You—Evidence-Based Contribution of Mechano-Biological Descriptors of Resistance Exercise

**DOI:** 10.3389/fphys.2021.686119

**Published:** 2022-01-05

**Authors:** Claudio Viecelli, David Aguayo

**Affiliations:** ^1^Institute of Molecular Systems Biology, ETH Zurich, Zurich, Switzerland; ^2^Kieser Training AG, Zurich, Switzerland

**Keywords:** resistance exercise, descriptors, mechano-biological, evidence-based, contribution

## Abstract

Skeletal muscle is one of the most important tissues of the human body. It comprises up to 40% of the body mass and is crucial to survival. Hence, the maintenance of skeletal muscle mass and strength is pivotal. It is well-established that resistance exercise provides a potent anabolic stimulus to increase muscle mass and strength in men and women of all ages. Resistance exercise consists of mechano-biological descriptors, such as load, muscle action, number of repetitions, repetition duration, number of sets, rest interval between sets, frequency, volitional muscular failure, and range of motion, which can be manipulated. Herein, we discuss the evidence-based contribution of these mechano-biological descriptors to muscle mass and strength.

## Introduction

Mechanically, skeletal muscle's function is to convert chemical energy into mechanical energy which can be used for force production and thus allows for locomotion. Metabolically, skeletal muscles are a sink for substrates such as amino acids, carbohydrates, fatty acids, minerals and inorganic salts. Moreover, they help maintaining the basal energy metabolism besides fueling muscle fibers during physical activity and/or exercise. Therefore, skeletal muscle mass regulates metabolic homeostasis and contributes significantly to survival (Fuhrman et al., 1951; Kotler et al., 1989).

Resistance exercise (RE) challenges the mechanical integrity (Ingber, 2003a,b) and metabolic homeostasis (Goto et al., 2005; Schoenfeld, 2013) of muscles. Systematically imposing mechanical and metabolic stress on the human body by RE leads to morphological and neural adaptations (Folland et al., 2007). These adaptations include e.g., changes in muscle fiber size (McDonagh and Davies, 1984; Jones et al., 1989) and architecture (Franchi et al., 2017), myofibrillar growth and mitochondrial proliferation (Macdougall et al., 1979; MacDougall et al., 1980), metabolic profile (Zanuso et al., 2017), tendon stiffness and hypertrophy (Reeves et al., 2003; Kongsgaard et al., 2007), firing frequency (Leong et al., 1999), cortical adaptations (Perez et al., 2004), spinal reflexes (Aagaard et al., 2002), and antagonist coactivation (Baratta et al., 1988). Additionally, muscles play, *de facto*, an endocrine role as they are able to release myokines, which have the capacity to interact in a hormone-like fashion and are involved in organ crosstalk, including muscle-liver, muscle-adipose tissue and muscle-bone crosstalk (Pedersen and Febbraio, 2012; Whitham and Febbraio, 2016). Muscle contractions, as seen during RE, stimulate the endocrine function of muscle cells (Bay and Pedersen, 2020). RE is associated with improved blood pressure control (MacDonald et al., 2016), improved insulin sensitivity controlling blood glucose (Codella et al., 2018), weight management (Paoli et al., 2015), depression management (Gordon et al., 2018; Pedersen, 2019), and bone mineral density (Zhao et al., 2015). Therefore, RE is considered medicine (Westcott, 2012).

However, despite receiving significant experimental attention, efficient and effective manipulation of RE mechano-biological descriptors remain under constant debate. It was the aim of this work to review the evidence of the contribution to strength and muscle mass of mechano-biological descriptors of RE.

## Mechano-Biological Descriptors of Resistance Exercise

### Load and Intensity

RE load is referred to the mass of an object (e.g., dumbbell) in SI-units [kg] or RE intensity, which is commonly defined as the percentage of maximal strength [% 1-repetition maximum (1-RM)] (Stone and O'Bryant, 1987; O'Bryant, 1997; Fleck and Kraemer, 2004). Mechanical stress, applied through the external loading of muscle fibers, is one of the primary mechanisms attributed to muscle mass accretion in response to RE (Schoenfeld, 2010). Therefore, the hypertrophic response to RE seems to be driven by mechanical tension (Goldberg et al., 1975; Fry, 2004).

Regarding intensity, moderate to high intensity (>60% 1-RM) is commonly recommended to induce gains in strength and muscle mass (ACSM, 2009). Up to date, it is inherently believed that the higher the loading, the bigger the muscle growth (Burd et al., 2012). However, although RE with high loads is effective in inducing hypertrophy and strength (Rhea et al., 2003), the latest research in untrained (Mitchell et al., 2012; Assunção et al., 2016; Lasevicius et al., 2018), and trained (Morton et al., 2016) adults consistently revealed that the RE load does not influence hypertrophy or strength development.

In a study, trying to remove the influence of an external load and to determine if muscle growth can be stimulated by solely maximally voluntary contracting the muscle through a full range of motion (ROM), Counts et al. (2016), subjected 13 participants to 18 sessions of unilateral elbow flexion exercise. Each arm was assigned to either the no load or high load (70% 1-RM) condition in a counterbalanced design. For the no load condition, participants solely repeatedly contracted dynamically as hard as possible through the full ROM with no external load. The authors reported significant differences between the two conditions for the post-intervention assessment of the elbow flexor 1-RM. Although 1-RM increased in both conditions post-intervention, the increase in the high load group was significantly higher (*P* = 0.032) than in the no load group. Muscle thickness measured by ultrasound (US), was increased in both groups when comparing pre- to post-intervention measurements and showed no significant difference between the groups. However, these results must be interpreted with caution. Firstly, the 1-RM test was highly specific, nonetheless pointing toward the need of higher loads for strength gains primarily, and secondly, US measurements are unidimensional and have far less relevance for detecting muscle hypertrophy (Scott et al., 2021).

Kumar et al. (2009), examined the relationship between myofibrillar protein synthesis (MPS) and anabolic signaling by work-matched RE at 20 – 90% of 1-RM in young (24 ± 6 years, n = 25) and old (70 ± 5 years, *n* = 25) men with identical body mass indices (24 ± 2 kgm^−2^). MPS was measured by stable-isotope tracers 1, 2, and 4 h post-exercise. They found that intensity and MPS were related in a sigmoidal dose-response relationship, whereas intensities >60% 1-RM plateaued. Elderly men showed a diminished response in comparison with young adults.

However, non-fatiguing protocols and their outcomes must be interpreted with caution. It was demonstrated, that in resistance-trained young men (21 ± 1 years, *n* = 15), lower intensity (30% of 1-RM) and higher volume RE performed until muscular failure was equally effective in stimulating MPS rates during 0 – 4 h recovery as heavy intensity (90% of 1-RM) and lower volume RE. Interestingly, exercise performed at 30% of 1-RM to muscular failure induced a longer-lasting effect on MPS at 21 – 24 h of exercise recovery (Burd et al., 2010).

Mechanical stress to the point of concentric muscular failure, regardless of the load or intensity, has been postulated to ultimately result in full-spectrum muscle fiber recruitment of all motor units available for this motor task (Carpinelli, 2008; Burd et al., 2012). Therefore, it is fair to conclude that external load might be of subordinate role concerning the MPS if RE is performed to muscular failure. Hence, a practical recommendation would be: the lower the load applied, the more important muscular failure of a motor task becomes.

### Muscle Action

Force generation, e.g., by muscle contraction, is achieved between actin and myosin filaments according to the sliding filament theory of skeletal muscle (Podolsky and Schoenberg, 1983).

Muscle action refers to the distinct biomechanical properties of actin-myosin filaments during muscular work. An activated muscle produces force. Actin-myosin filaments can either slide towards (concentric) or apart (eccentric) each other or not slide at all (isometric). To describe these different ways of muscle action, the terms concentric, eccentric, and isometric contractions were introduced (Wall and Karpovich, 1960).

Mechanistically, concentric, eccentric, and isometric muscle contractions can be distinguished by their ability to generate force. Eccentric force generation is associated with 20–50% greater strength in comparison to concentric force generation (Petrella et al., 2006). Metabolically, eccentric work is associated with about a 6-fold decreased energy cost (Hoppeler, 2016) which opens up unique RE opportunities for clinical populations (Hoppeler, 2014). Hence, eccentric contractions are more efficient than concentric contractions.

As eccentric contractions have a higher force capacity than concentric or isometric contractions, it is assumed that the increase in mechanical tension would promote greater hypertrophy and strength (Roig et al., 2009). Assigning single hypertrophic contributions to either form of contraction is difficult as muscle activation, recruitment, force capacity, and metabolism are distinct between the contraction modes. As such, it is obvious that the same external loading is leading to different work volumes and intensities. In consideration of the aforementioned discrepancies, Carey Smith and Rutherford (1995) used a within-subject study design whereby five males (20.6 ± 0.9 years) and five females (20.2 ± 1.3 years) trained one leg using concentric and the other leg using eccentric contractions of the *quadriceps* muscle for 20 weeks. The loading for the eccentric condition was 35% higher than for the concentric condition. The cross-sectional area (CSA) was assessed using computed tomography (CT). Significant muscle mass increases were found from pre- to post-intervention in both groups (*P* <0.05). However, no significant difference was reported between the two legs. Numerous studies investigating muscle action using direct-site measurements (Carey Smith and Rutherford, 1995; Blazevich et al., 2007; Reeves et al., 2009; Moore et al., 2012; Farup et al., 2014; Franchi et al., 2014; Mamerow et al., 2014), lean body mass (Nickols-Richardson et al., 2007), and circumference (Cadore et al., 2013) of muscle hypertrophy in untrained and trained individuals reported no significant superiority of either contraction mode. In summary, a clear indication for a superior effect of a concentric *versus* eccentric, or *vice versa* muscle action modality on muscle hypertrophy, has not been observed so far (Mayhew et al., 1995; Higbie et al., 1996; Hortobágyi et al., 1996, 2000; Seger et al., 1998; Farthing and Chilibeck, 2003; Vikne et al., 2006; Farup et al., 2014).

Multiple studies addressed isometric training. Jones and Rutherford (1987) compared the three contraction forms in a within-subject design whereby 12 untrained individuals (11 males, one female, 27.5 ± 6 years) were recruited. Six individuals (five males, one female) trained one leg with concentric contractions (80% 1-RM, 2 – 3 s per repetition) and the other leg with eccentric contractions (145% 1-RM, 2 – 3 s per repetition) on a leg extension machine. Six subjects used unilateral isometric contractions and the contralateral leg served as control. For 12 weeks, the maximum isometric force of the *quadriceps* was tested weekly, whereby the training target was set to 80% maximum voluntary contraction. A strength-testing chair was used where each contraction was held 4 s with 2 s rest between. For all contraction forms, substantial increases in *quadriceps* force and hypertrophy were found, whereas no significant differences between the contraction forms (*P* > 0.05) were reported.

Kubo et al. (2001) addressed the influence of isometric contraction durations on the elasticity of human tendon structures *in vivo*. Therefore, eight young men (22.6 ± 2.8 years) were recruited and a within-subject design was used whereby one leg was assigned to a long- and the other leg to a short-duration contraction protocol. The long-duration protocol consisted of four sets of four contractions and contraction for 20 s and relaxation for 60 s. The short-duration protocol used three sets of 50 repetitions of contraction for 1 s and relaxation for 2 s. 70% of the maximum voluntary contraction was used. The experimental period was 12 weeks with a training frequency of 4-days per week. Muscle volume was calculated using MRI. Both protocols induced significant increases in maximum voluntary contraction force and muscle volume without significant differences between the training protocols (*P* > 0.05).

Hypertrophy, driven by mechanical and metabolic stress, can be induced by maximizing the fiber recruitment—time integral. It is obvious that this can be achieved using concentric, eccentric and isometric muscle contractions although activation, recruitment, force capacity, and metabolism are distinct (Franchi et al., 2017). Current literature does not allow for drawing any conclusions on the superiority of either of the muscle actions. As such, the use of all forms of muscle actions is recommended for increasing strength and hypertrophy (ACSM, 2009).

### Number of Repetitions

In RE, a repetition is defined as the complete cycle of lifting and lowering a mass over a defined ROM of a specific exercise. The American Colleague of Sports Medicine (ACSM) published multiple position stands concerning recommendations for RE, with repetition recommendations ranging from 8 to 12 repetitions for skeletal muscle hypertrophy (Kraemer et al., 2002; ACSM, 2009). The National Conditioning Strength Association (NCSA) recommends 6–12 repetitions for inducing muscle growth and <6 repetitions for inducing strength (Gregory Haff and Travis Triplett, 2016). Hence, repetition numbers are one of the most varied RE descriptors.

The generation of force is directly associated with energy consumption, indicating a role for metabolic stress (i.e., accumulation of metabolites during exercise) in RE. Metabolic stress is recognized as an important driver in the development of muscle mass and strength (Rooney et al., 1994; Carey Smith and Rutherford, 1995; Schott et al., 1995; Goto et al., 2005; Schoenfeld, 2013; Popov et al., 2015; Ozaki et al., 2016). Mechanical work (i.e., the number of repetitions-time integral) can be considered as the accumulated metabolically induced stress or the expended energy over time. Therefore, energy expenditure and metabolic stress are increased in high-repetition RE to muscular failure when compared to low-repetition RE.

Brunelli et al. (2019) investigated the energy expenditure (EE) during and after an acute low-load and high-repetition or high-load and low-repetition resistance training protocol in young adults (22 ± 3 years, *n* = 11) in a randomized crossover design of three sets of non-work-matched knee extensions using 30% 1-RM with an average repetition number of 27 and 80% 1-RM with an average repetition number of eight repetitions to muscular failure. The exercise EE in the 30% 1-RM group was significantly higher when compared to the 80% 1-RM group (*P* = 0.0243). Plasma lactate levels in the 30% 1 RM group were also significantly higher 3-, 5-, and 7-min post-exercise indicating the energy contribution of the anaerobic lactic system which is in line with the higher number of repetitions performed (*P* = 0.0001).

Wernbom et al. (2007) quantified the dose-response relationship between training variables and hypertrophy in a systematic review and found that the average increase rate of CSA for the elbow flexors increased 0.15, 0.26, and 0.18% per day for repetition ranges of 7 – 38 repetitions, 42 – 66 repetitions, and 74 – 120 repetitions per exercise and session, respectively.

As described above, numerous recommendations for the number of lifting repetitions exist (Kraemer et al., 2002; ACSM, 2009; Gregory Haff and Travis Triplett, 2016). Repetition numbers, empirically and scientifically tested, are completely arbitrary numbers as low (high-intensity) and high (low-intensity) numbers of repetitions can induce muscle hypertrophy and strength (Morton et al., 2016). Additionally, it must be emphasized that muscle does not possess any internal counting mechanisms. On the contrary, muscle contractions are solely energy-dependent, which, in turn, is a function of substrate supply (i.e., metabolic enzymes, energy availability, etc.), and demand (i.e., intensity). Thus, if high level of metabolic stress and resulting fatigue is achieved more muscle fibers will be recruited to sustain force output. Thus, if RE is performed to muscular failure, regardless of the repetition number, the hypertrophic stimulus will likely be similar.

### Repetition Duration

The time it takes to complete a single repetition is referred to as repetition duration. The duration is often communicated with three or four digits numbers, one for each mode of action (i.e., concentric, eccentric, and isometric) in seconds (Schoenfeld, 2016). The single repetition duration is dependent on the velocity, which in turn is dependent on the externally applied load and fatigue. It is well-established that force is inversely proportional to velocity (Hill, 1938). Hence, an increase in load and therefore in force production will inevitably decrease velocity (Sánchez-Moreno et al., 2017). Additionally, the more fatigue is accumulated over preceded repetitions, velocity will decrease because muscle fibers are unable to maintain a constant force output, mainly driven by transient increases in free [ADP] in the myoplasm (Allen et al., 2008).

A systematic review and meta-analysis, including eight studies with 239 individuals, analyzed how repetition duration affected the hypertrophic response to resistance training performed to muscular failure (Schoenfeld and Ogburn, 2015). Repetition duration was stratified as follows: fast heavy (sets of 6 – 12 repetition with total repetition 0.5 – 4 s), fast light (sets of 20 – 30 repetitions with a total repetition duration of 0.5 – 4 s), medium (sets of 6 – 12 repetitions with a total duration 4 – 8 s), or slow (sets of 6 – 12 repetitions with total repetition duration of > 8 s). The effect sizes were 0.67 ± 0.19, 0.79 ± 0.37, 0.27 ± 0.20, and 0.29 ± 0.27 for the fast/heavy, fast/light, medium and slow group, respectively. A sub-analysis of direct measures of muscle hypertrophy (magnetic resonance imaging (MRI), US and muscle biopsy), including five out of eight studies, revealed an effect size 0.42 ± 0.17 for the fast/heavy duration and 0.37 ± 0.17 for the medium duration, respectively. Although the mean effect sizes of fast/heavy and fast/light (~0.73) in comparison to medium and slow (~0.33) would suggest a superiority in faster speed groups, confident intervals were wide for all groups indicating large within-groups variances and therefore the results must be interpreted with caution.

Morton et al. (2019) investigated muscle fiber activation using biopsies in a study where light and heavy loads were lifted to muscular failure with normal and longer repetition duration. Ten recreationally trained young men (23 ± 1 years) performed four unilateral REs. The groups consisted of exercises at 80% 1RM at regular and slow and 30% 1RM regular and slow movement velocities. Each participant performed two of the four conditions consecutively (one on each leg), which involved three sets to motor task failure. Repetition duration per set differed significantly between all groups (*P* < 0.05) while the regular group and the slow group did not differ significantly concerning total training volume (*P* > 0.05). Repetition duration or varying loads did not result in significant differences in fiber type-specific glycogen depletion (*P* > 0.20) which is a direct measure of the fiber use and hence of the proceeding activation (Gollnick et al., 1973, 1974; Kopke et al., 1984; Vollestad and Blom, 1985) when RE is performed to failure. Hence, no matter of the repetition duration or the external load, the full spectrum of fibers is recruited for the motor task.

The literature suggests that a wide range of isotonic repetition durations can be used ranging from fast (0.5 s) to slow (8 s) to effectively induce anabolic signaling and therefore strength and hypertrophy. However, the recruitment of the full muscle fiber spectrum by performing RE to failure stimulates hypertrophy rather than any arbitrary repetition duration.

### Number of Sets

A set is the number of cycles of repetitions performed. Like for the number of repetitions, numerous recommendations for the numbers of sets exist, ranging from 2 to 6 sets for maximal muscle hypertrophy and strength (Kraemer et al., 2002; ACSM, 2009; Gregory Haff and Travis Triplett, 2016). To estimate and compare RE protocols, the term exercise volume load was established, reflecting lifted load × number of sets × number of repetitions.

Krieger (2010) compared the effects of single to multiple sets on hypertrophy using a multilevel meta-regression, including eight studies with 322 individuals. The effect size of multiple sets was found to be 44% greater performing multiple sets (ES: 0.35 ± 0.03) in comparison to the performance of a single set (ES: 0.25 ± 0.03) (*P* < 0.05). Noteworthy, the effect sizes increase with the number of sets performed as follows: 0.24 for 1 set, 0.34 for 2 – 3 sets, and 0.44 for 4 – 6 sets. Regarding strength, the effect size of performing multiple sets (ES: 0.80 ± 0.11) compared with a single set (ES: 0.54 ± 0.11) was 33% greater (*P* < 0.0001) (Krieger, 2009).

Wernbom et al. (2007), quantified the dose-response relationship between training variables and hypertrophy in a systematic review and found that the average increase rates of CSA for the elbow flexors, for total sets analyzed, were 0.17, 0.24, and 0.18% for 3 – 3.5 sets, 4 – 6 sets, and ≥ 9 sets, respectively.

In a study researching the response of performing 1, 3, and 5 sets on muscle strength and hypertrophy, 48 untrained men (24.4 ± 0.9 years) were randomly assigned to one of the three training groups whereas all groups performed three weekly resistance training sessions for 6 months (Radaelli et al., 2015). Muscle thickness for the elbow flexors was measured using US and effect sizes of 0.10, 0.73, and 1.10 were found for 1 set, 3 sets, and 5 sets, respectively. Both 3 and 5 sets groups showed a significant increase in muscle thickness when compared with the 1 set group (*P* ≤ 0.05). Five sets were associated with a significantly greater increase than 3 sets (*P* ≤ 0.05). For the elbow extensors thickness, effect sizes of 0.05, 0.05, and 2.33 for 1 set, 3 sets, and 5 sets were reported. Only the 5-set group increased muscle thickness of the elbow extensor significantly (*P* ≤ 0.05), indicating a volume dependence.

Schoenfeld et al. (2019) examined hypertrophy and strength in trained men by using three resistance training protocols differing by volume. Thirty-four males (23.8 ± 3.8 years) were randomly allocated to three groups, a low-volume group, a moderate-volume group and a high-volume group, respectively. While the low-volume group performed 1 set per training session, the moderate-volume group performed 3 and the high-volume group 5 sets per training session. Three weekly sessions were conducted over an eight-week period. For the assessment of strength, squat and bench press 1-RM was evaluated while US was used to verify morphological changes of the elbow flexors and extensors, mid-thigh and lateral thigh. No significant between-group differences were detected for the squat (*P* = 0.22) and bench press (*P* = 0.15) 1-RM. Elbow flexor thickness (*P* = 0.02), mid-thigh *m. rectus femoris* thickness (*P* = 0.02), and lateral thigh *m. vastus lateralis* (*P* = 0.006) were significantly different when comparing 1 set to 5 sets in favor of 5 sets. No significant difference was reported between the groups for the improvement of the *m. triceps* (*P* = 0.19) thickness.

In contrast, Mitchell et al. (2012) randomly allocated the legs of 18 untrained men (21 ± 1 years) to training conditions differing in intensity (% of 1-RM) or volume (1 or 3 sets of repetitions): 30%-3, 80%-1, and 80%-3. Participants exercised each leg to failure with the assigned condition thrice a week for 10 weeks. MRI was used to detect changes in muscle mass from pre- to post-measurements. The training-induced muscle volume increases were significant for all groups (*P* = 0.01). However, no significant difference between the groups (*P* = 0.18) was detected. The authors reported that strength gains did not differ between the high-intensity groups but were significantly greater than in the 30%-3 group (*P* = 0.04).

Ribeiro et al. (2015) examined strength and hypertrophy in 32 randomly allocated elderly women after 12 weeks of performing 1 or 3 sets per session of full-body resistance training. Dual-energy x-ray absorptiometry (DXA) was used to assess hypertrophy. Strength was evaluated using 1-RM in chest press and knee extension. The group performing 3 sets increased strength more significantly (*P* < 0.05*)*. No significant differences were reported for hypertrophy between the groups (*P* > 0.05).

Exercise volume, a variable easy to manipulate by the increase of the number of sets, seems to influence the hypertrophic response in untrained and trained individuals. However, caution should be used when generalizing this result as the vast majority of studies are carried out in untrained subjects and/or young, trained males. In addition, as accumulated time-under-tension (TUT) significantly differs and thus metabolic stress, results must be interpreted carefully. As apparent from the effect size figures for the CSA rate increase for the elbow flexors from Wernbom et al. (2007) and Ogasawara et al. (2017), an inversely u-shaped relationship between volume and hypertrophy might exist whereby further increases in volume seem to diminish hypertrophic responses (Helms et al., 2014).

### Rest Interval Between Sets

The time spent between starting the next set is referred to as rest interval or rest period. Rest intervals reflect the trade-off between mechanical tension and metabolic stress. Hence, the length of rest intervals is negatively correlated with either mechanical tension or metabolic stress. The recommendations of the ACSM (ACSM, 2009) for the length of rest intervals ranges from 1 to 2 min for 6 – 12 RM, 3 – 5 min for power training with fast contraction speeds and <90 s for local muscular endurance training with light to moderate loads (>15 repetitions).

Ratamess et al. (2007) examined the effects of different rest intervals on metabolic responses to the bench press. Eight resistance-trained men (21.4 ± 2.4 years) performed 10 randomized protocols (five sets of bench press with 75 or 85% of 1RM for 10 repetitions and five repetitions, respectively) using different rest interval length (30 s, 1, 2, 3, 5 min). Oxygen consumption (VO_2_) was measured during and up to 30 min post-exercise. The major findings were that RE volume was reduced proportionally as rest intervals were reduced for both intensities. Short rest intervals (30 s and 1 min) elicited higher post-exercise oxygen consumption response during five repetitions but had no substantial effect during 10 repetitions. The acute metabolic response pattern was similar between both intensities, however, the area under the curve of the VO_2_ magnitude was significantly higher in the 10-repetition group (*P* < 0.05). The fatigue rate was significantly correlated to the metabolic response to acute RE (*P* < 0.001). 30 s rest interval decreased total training volume by more than 24% in the 10-repetition group in comparison to the group that had 5 min rest interval.

Ahtiainen et al. (2005) addressed the question of short and long rest intervals on strength, muscle size and hormonal adaptations of the *quadriceps* muscle in trained men. Thirteen recreationally strength-trained individuals (28.7 ± 6.2 years) participated in the crossover-design study. Two training groups underwent either a short-rest (2 min) or a long-rest (5 min) 3-month crossover-intervention. The training protocol consisted of five sets of leg presses, four sets of squats and 10 repetitions per set for the short-rest group. The long-rest group did four sets of leg presses, three sets of squats and 10 repetitions. Additionally, load in the long-rest group was increased (~15%) to adjust for total volume. Bilateral isometric leg extension force did not differ significantly between the groups at time-point 0, 3, or 6 months. The CSA of the *m. quadriceps femoris*, measured by MRI, increased significantly by 3.5 ± 4.3% after 6 months (*P* < 0.05) but not significantly after 3 months in both groups (*P* > 0.05).

Without controlling for total volume, de Souza and colleagues (De Souza et al., 2010) investigated muscle strength and hypertrophy between constant and decreasing rest intervals using 8 weeks of resistance training. Twenty recreationally trained, young men (20.5 ± 1 years) were randomly assigned to either a constant or a decreasing rest interval training group. During the first 2 weeks of training, three sets of 10 – 12 repetitions with 2-min rest intervals in-between sets and exercises were performed by both groups. During the coming 6 weeks, the constant rest interval group used 2 min between sets and exercises (four sets of 8 – 10 RM) and the decreasing rest interval group trained with rest intervals decreasing from 2 min to 30 s (four sets of 8 – 10 RM). Hence, the total training volume of the bench press and squat were significantly lower for the decreasing compared to the constant rest interval group (bench press 9.4%, squat 13.9%, *P* = 0.043). Muscle CSA was assessed using MRI. No significant differences were reported between the two groups for the CSA (arm 13.8 vs. 14.5%, thigh 16.6 vs. 16.3%), 1RM (bench press 28 vs. 37%, squat 34 vs. 34%) and isokinetic peak torque (*P* ≤ 0.05).

Schoenfeld et al. (2016), examined resistance training adaptations in 21 young resistance-trained men (range: 18 – 35 years) that were randomly assigned to either a group that used 1-min rest intervals or a group that used 3-min rest intervals. Total training volume was equated. The study period lasted 8 weeks and the participants were performing three total body workouts a week comprising three sets of 8 – 12 repetition of seven different exercises per session. Muscle strength was tested using 1RM bench press and back squat while muscle thickness of the elbow flexors, *m. triceps brachii* and *m. quadriceps femoris* were assessed using US. A significantly greater increase for 1RM bench press (*P* = 0.02) and squat (*P* < 0.01) for the group using the 3-min rest interval in comparison to the 1-min rest interval group was reported. Although not significantly different, a trend was noted for greater increases in the *m. triceps brachii* (*P* = 0.06) thickness for the longer rest interval condition.

In summary, rest intervals impact total training volume and are a trade-off between mechanical tension and metabolic stress. However, current literature suggests that rest intervals have little effect on strength and hypertrophy when controlled for total training volume.

### Frequency

The number of exercise sessions performed per muscle group, generally per week, is referred to as (exercise) frequency (Schoenfeld, 2010). Based on the training status, the NASC (Gregory Haff and Travis Triplett, 2016), recommends 2 – 3, 3 – 4, and 4 – 7 exercise sessions per week for beginners, intermediate and advanced individuals, respectively. The ACSM recommends 2 – 3 and 4 – 5 exercise sessions for novice, intermediate and advanced individuals, respectively (Kraemer et al., 2002).

A recent systematic review and meta-analysis including 22 studies revealed that exercise frequency showed a significant effect on muscular strength gains, whereby effect sizes increased in magnitude from 0.74, 0.82, 0.93, and 1.08 for training one, two, three, and four or more times per week, respectively (Grgic et al., 2018a). However, subgroup analysis of volume-equated studies and training to muscular failure studies revealed no significant effect of frequency on muscular strength (*P* = 0.421). Interestingly, age and sex showed significant effects of frequency on strength. While in the middle-aged and elderly adults group effect sizes increased in magnitude with each additional exercise session, the linear trend was not significant (*P* = 0.093). The effect sizes gradually increased in magnitude in the young group with each additional exercise session per week with a significant overall effect of training frequency (*P* = 0.024). Regarding sex, while the effect of frequency on strength was not significant for men (*P* = 0.19) it was found to be significant for women (*P* = 0.03).

In the same year, Barcelos et al. (2018) confirmed the observations that frequency has no effect on strength nor muscle CSA if RE is performed to muscular failure. In their study, they used a within-subjects design in which 20 untrained males (23 ± 4 years) had one leg randomly assigned to a frequency of 2-, 3-, or 5 times weekly strength training which consisted of 9 – 12 repetitions to muscular failure at 80% 1RM on a leg extension machine for eight weeks. Although total training volume was significantly different between groups (*P* < 0.05), increases in strength (*P* > 0.05) and *m. vastus lateralis* CSA (*P* > 0.05) did not differ significantly between groups. The effect sizes for 1RM at week 8 were 1.98, 1.40, and 1.81 for 2-, 3-, and 5-times weekly training sessions, respectively. Concerning muscle hypertrophy, the effect sizes for CSA were found to be 0.7, 0.58, and 0.69 at week 8 for 2-, 3-, and 5-times weekly training sessions, respectively.

Grgic et al. (2018b), reviewed existing literature to examine the association of RE frequency and hypertrophy. Using studies that used direct site-specific measures of hypertrophy, they reported that resistance training once a week elicits similar hypertrophy compared to training two or three times per week. Comparing studies using lean body mass for the estimation of muscular growth, no significant differences between training frequencies were found too. Grgic et al. concluded that under volume-equated conditions, the frequency seems not to have a pronounced effect on hypertrophy.

In a weekly volume-matched approach, Gomes et al. (2019) examined resistance training frequency in increasing muscle mass and strength in well-trained men. Twenty-three subjects (26.2 ± 4.2 years) were randomly allocated into two groups: low-frequency or high-frequency resistance training. The low-frequency group performed a split-body routine, training each specific muscle group once a week while the high-frequency group trained all muscle groups every session five times per week. Strength was measured using squat and bench press 1RM and lean tissue mass was measured using DXA. It was reported that both groups improved muscle strength (*P* < 0.01) and muscle mass (*P* = 0.007) when comparing pre- to post-intervention. However, no significant difference between groups for strength and muscle mass was found.

Kneffel et al. (2020) examined resistance training frequency in adults over 60 years of age on muscular strength and hypertrophy in a recently published meta-regression. Fifteen studies were analyzed including 597 individuals of both sexes. The authors showed that upper- and lower body strength improvements were dependent on the number for training days but frequency had no effect on hypertrophy in adults over 60 years (*P* = 0.67).

In summary, frequency seems not to have an influence on strength or hypertrophy if training is performed to muscular failure even though total training volume significantly differs. It is generally recommended allowing at least 48 h of recovery between RE sessions for the same muscle group (Schoenfeld, 2010) as it allows for the MPS to fully unfold (MacDougall et al., 1995).

### Volitional Muscular Failure

Volitional muscular failure is the neuromuscular inability to complete a concentric contraction over the full ROM (Schoenfeld, 2010; Sampson and Groeller, 2016; Steele et al., 2017). Training to failure is thought to achieve full motor unit recruitment, a possible premise for increasing muscle size (Wernbom et al., 2007). When the external load is high (≥85% 1-RM), higher threshold motor units are immediately recruited to provide high force production while lower loads do not immediately recruit higher threshold motor units as high muscle force production is not needed initially (de Luca and Contessa, 2012). With increasing neuromuscular fatigue, higher threshold motor units are gradually recruited to compensate for motor units that could not sustain force generation (Adam and De Luca, 2005). Consequently, by reaching volitional muscular failure, the complete available motor unit pool for the corresponding motor task was recruited (Adam and De Luca, 2005). As such, volitional muscular failure plays an increasingly important role when the training load is reduced. Additionally, training to failure may also influence muscular growth *via* increased metabolic stress (Schott et al., 1995), contributing to anabolic signaling.

Mitchell et al. (2012) showed in untrained individuals (21 ± 1 years, *n* = 18) that a plethora of work intensities (i.e., 30 vs. 80% of 1-RM) executed to muscular failure can be applied to induce muscle hypertrophy of the *m. quadriceps*. Using different contraction intensities (i.e., 30 vs. 80% of 1-RM) and training volumes (i.e., 1 or 3 sets of repetitions) three times weekly for 10 weeks of knee extension, induced significant hypertrophy in all groups (*P* < 0.001) while no significant difference between the 80%-1 and 80%-3 group (*P* = 0.18) was found. Strength gains did not differ between the high-intensity groups but were significantly greater than in the 30%-3 group (*P* = 0.04).

Morton et al. (2016) showed in trained individuals using a full-body resistance training that load does not determine resistance training-mediated hypertrophy or strength gains. Forty-nine trained men (23 ± 1 years) were recruited and trained for 12 weeks. Individuals were randomly allocated into a higher- or lower-repetition group lifting either ~30 – 50% or ~75 – 90% 1-RM for 20 – 25 repetitions per set or 8 – 12 repetitions per set, respectively. Participants performed all sets to failure. Strength, determined by 1-RM, increased for all exercises in both groups (*P* < 0.01), except for the bench press, being different between groups (*P* = 0.012). Type 1 and type 2 muscle fiber CSA increased following training (*P* < 0.01) with no significant differences between groups (*P* > 0.05). The authors concluded that, in resistance-trained individuals, when exercises are performed to volitional failure, the load does not dictate hypertrophy or, mostly, strength gains.

In contrast, Sampson and Groeller (2016) found similar adaptations across three different intense resistance training protocols, suggesting repetition to muscular failure is not critical for neural and structural changes of skeletal muscle. They subjected 28 males to a 4-week familiarization. Afterwards, according to their increases in strength gains measured by 1-RM, subjects were counterbalanced into three groups: non-failure rapid shortening (fast concentric, 2 s eccentric), non-failure stretch-shortening (fast concentric, fast eccentric), and failure control (2 s concentric, 2 s eccentric). The experimental period lasted 12 weeks and comprised unilateral elbow flexor resistance training using 85% 1-RM 3 times weekly. While the failure control group performed repetitions to failure in all 4 sets, participants in the non-failure groups completed only four repetitions per set. 1-RM, maximal voluntary contraction and muscle CSA increased as determined by MRI. No differences between groups were detected (*P* > 0.05), suggesting repetition to muscular failure is not a premise.

Nóbrega et al. (2018) examined resistance training where participants either had to perform to muscular failure or to the point where participants volitionally interrupted the exercise. The interventional period was 12 weeks and a within-subject design was applied, whereby 32 untrained men (23.0 ± 3.6 years) performed unilateral leg extension twice a week. The legs of participants were randomly allocated according to 1-RM and CSA values to either a high-intensity to failure (i.e., 80% 1-RM) or high-intensity to volitional interruption (i.e., 80% 1-RM), a low-intensity to failure (i.e., 30% 1-RM) or a low-intensity to volitional interruption (i.e., 30% 1-RM) group, respectively, each performing 3 sets. Muscle CSA and 1-RM were not different between groups (*P* > 0.05) following 6- and 12-weeks post-intervention. The authors suggested that all protocols were similarly effective in inducing hypertrophy and strength, indicating an increased efficiency for training close to failure. Caution must be given, as the volume loads of the intervention groups were similar, being a possible confounder.

Neves et al. (2018) investigated adaptations to concurrent training in elderly men. The experimental period lasted for 12 weeks and consisted of strength training always prior to endurance training within the same training session twice a week. As the *quadriceps* muscles were investigated, RE comprised leg press and bilateral knee extension. Fifty-two elderly men (66 ± 5 years) were recruited and distributed into three groups. One group performed 2 – 3 sets of repetitions to failure at 70% 1-RM, a group that performed 2 – 3 sets of 8 – 10 repetitions not to failure and a group that performed 8 – 10 repetitions not to failure at 50% 1-RM, but with volume equalized to the failure group. *Quadriceps* muscle thickness was assessed using US and showed that the intervention elicited similar *quadriceps* muscle thickness increases in the group that performed the repetitions to failure and the volume-equalized group. Age should therefore be of consideration when prescribing RE performed to muscular failure.

In summary, the results from the cited studies show that lower loads can be equally effective as higher loads if RE is performed to muscular failure. However, the topic is still ambiguous as some studies showed benefits for accomplishing muscular failure during dynamic RE (Rooney et al., 1994; Drinkwater et al., 2005; Schoenfeld et al., 2015) while others reported no benefit (Sampson and Groeller, 2016; Martorelli et al., 2017; Nóbrega et al., 2018).

Exercise volume and training to failure are equally effective in inducing hypertrophy and strength in untrained older men, while younger and RE-experienced individuals might benefit from RE to failure. As full muscle fiber recruitment can be achieved with high loads (i.e., > 80% 1-RM; de Luca and Contessa, 2012), reaching volitional muscular failure during high-load RE is not necessarily a premise in order to induce increases in functional and structural/morphological adaptations (Davies et al., 2016). In contrast, when applying lower load (i.e., ≤ 50% 1-RM) reaching volitional muscular failure seems to be essential for increases in muscle strength and mass. Hence, a practical recommendation would be: the lower the load applied, the more important the intensity of effort and, therefore reaching volitional muscular failure of a motor task becomes. Finally, performing RE to volitional muscular failure helps to ensure the delivery of an effective stimulus.

### Range of Motion

Range of motion (ROM) is defined as the extent of a performed movement over a joint. The 2009 ACSM (ACSM, 2009) position stand does not give any practical recommendations for the application of a ROM to elicit structural or functional muscular adaptations. Skeletal muscles force increases as a function of muscle length, plateaus, and then decreases (Gordon et al., 1966). This indicates that there is a length-dependent muscle–force relationship, which is directly related to the extent of overlap between the myosin and actin filaments in the sarcomere. In the literature, training with partial or full ROM to optimally increase muscle mass is discussed ambiguously. It is suggested that performing repetitions over the full ROM maximize mechanical stress over the full range of fibers and thus stimulating hypertrophy to a greater extent than partial ROM (Fleck and Kraemer, 2004). On the other hand, using partial ROM allows maximizing external loading as repetitions are performed with optimal force-length relationship and thus eliciting greater hypertrophic adaptions (Sisco and Little, 1997).

McMahon et al. (2014) examined the relationship of ROM during resistance training on hypertrophy, subcutaneous fat, and strength. Twenty-six recreationally active participants (19.6 ± 2.6 years) were allocated in a full ROM (0° – 90°) and partial ROM (0° – 50°) knee flexion group and underwent 8 weeks of RT with a frequency of three times a week and 4 weeks of detraining. Lower limb exercises using 80% 1-RM were controlled using a goniometer. Ten participants were allocated to the control group (23.6 ± 2.4 years). Significant post-training differences (*P* < 0.05) existed in strength and CSA distally in favor of the full ROM group. Morphological and architectural adaptions in response to resistance training were found to be greater in the full ROM group, resulting in significant increases in strength in comparison to the partial ROM group. The authors conclude that from a practical point of view, full ROM should be aimed at resistance training, where increased muscle strength and size are the objectives. Moreover, ROM should not be compromised for greater external loading.

Pinto et al. (2012) compared partial ROM to a full ROM upper-body resistance training in on strength and hypertrophy in young men. Participants were randomly assigned to three groups either full ROM (0° – 130°, 0° = full elbow extension), partial ROM (50° – 100°), or control. The subjects (21.7 ± 3.5 years, *n* = 40) trained 2 days a week for 10 weeks using a periodized program. Elbow flexion strength was examined using 1-RM and hypertrophy was assessed by US. Both intervention groups increased elbow flexion 1-RM significantly (*P* < 0.05). Post-intervention, full ROM strength was significantly greater (*P* < 0.05) than for the partial ROM group. Average elbow flexor hypertrophy significantly increased for both training groups (*P* < 0.05). The authors suggested that strength and hypertrophy can be elicited using full and partial ROM training, however full ROM might lead to greater improvements in strength than partial ROM. In line with this, Bloomquist et al. (2013), investigated the manipulation of joint ROM during squat training and its effects on adaptations to strength training. Seventeen male participants (24.0 ± 4.5) were randomly allocated to 12 weeks of squat training using full ROM (0° – 120°) or partial ROM (0° – 60°). 1-RM was used to assess strength and muscle CSA was examined using MRI. Both protocols induced significant changes in strength from pre- to post-intervention (*P* < 0.05). Full ROM training resulted in superior increases in front thigh muscle CSA (*P* < 0.05) in comparison to partial ROM training. The authors concluded that full ROM resistance training elicited favorable adaptations on knee extensor muscle CSA and function compared to partial ROM. In contrast, Valamatos et al. (2018) determined the effect of a 15-week partial ROM resistance training on the *m. vastus lateralis* architecture and mechanical properties when the TUT was equalized. For this, they recruited 19 untrained male subjects (24.1 ± 4.4 years) and randomly assigned them to a control or training group. In the training group, the dominant and non-dominant legs were randomly allocated to either be trained with a full ROM (0° – 100°) or a partial ROM (0° – 60°) in an isokinetic dynamometer. Full ROM resistance training changed fascicle length (*P* < 0.001) and specific tension (*P* < 0.001) while partial ROM seemed to have a moderate effect on physiological CSA (*P* < 0.05) and torque adaptations *(P* < 0.05). The authors concluded that architectural and mechanical muscle adaptations are ROM dependent as fascicle length and specific tension is increased if greater ROM is used. Conversely, the restriction of the ROM to shorter lengths is associated with angle-specific strength adaptations and seems to promote greater physiological CSA.

A recent systematic review concluded that muscle length during isometric training is relevant as it was shown that training at longer muscle lengths stimulated hypertrophy to a greater extent in comparison to isometric training at shorter muscle length, suggesting an equally effective training of partial and full ROM if partial ROM training is performed at long muscle length (Oranchuk et al., 2019). In contrast, studies performed with dynamic RE reported no statistically significant difference between the increases for full and partial ROM muscle thickness (Pinto et al., 2012; McMahon et al., 2014).

In support of partial ROM, Goto et al. (2019) investigated if partial ROM RE is effective in inducing muscle hypertrophy and function. They hypothesized that partial ROM training induces higher vascular occlusion than full ROM training, mediated through greater muscular tension and constant contractions, resulting in increased hypertrophy and strength in resistance-trained men. Therefore, they recruited 44 men (20 – 22 years), allocating them into a partial ROM (45° – 90°) or a full ROM (0° – 90°) group whereby the elbow extensors were trained trice a week for 8 weeks. Participants had to perform three sets of 8 repetitions in each training session. Increases in CSA of *m*. *triceps brachii*, measured by US, and isometric strength were found for both groups (*P* ≤ 0.05), with CSA being significantly greater after partial than after full ROM (*P* ≤ 0.05) resistance training. The authors suggested that greater hypoxia, mediated through partial ROM training, might contribute to increased hypertrophic signaling.

In conclusion, it can be stated that full ROM seems to be beneficial for inducing muscle hypertrophy when compared to partial ROM exercise training. Moreover, it seems that some threshold exists, beyond more excursion does not bring additional benefits. Nonetheless, if partial ROM is applied, the focus should be placed on longer muscle lengths. Persons with musculoskeletal problems resulting in a diminished ROM might benefit, as some evidence implies that partial ROM resistance training can produce hypertrophic gains.

## Conclusion

This article synthesizes latest scientific evidence of resistance exercise mechano-biological descriptors. In summary, the evidence presented in this article shows that the discussion about load or intensity is overrated for a non-athlete population as high and low loads can induce significant gains in strength and hypertrophy if muscular failure is reached. Nonetheless, high loads seem likely to be necessary to maximize strength development. In addition, metabolic stress is also a potent driver in the development of muscle mass and strength. As described above, a wide range of repetition durations can be used to increase muscle mass and strength if muscle tension is continuously sustained and performed to failure. Exercise volume seems to influence the hypertrophic response in untrained individuals. Therefore, we suggest for this cohort to increase RE training volume by increasing the number of repetitions, and/or sets and/or frequency. The rest interval between single sets of resistance exercise to increase training volume seems not to have any effect on muscle mass or strength. To allow recovery between resistance exercise sessions, at least 24–48 h of recovery is recommended per muscle group. As current evidence does not allow to draw conclusions on the superiority of either concentric, isometric or eccentric muscle action, all forms should be used to increase muscle mass and strength. Resistance exercise to muscular volitional failure during high-load resistance exercise is not a premise to induce increases in functional and structural adaptations. In contrast, when applying lower load (i.e., ≤ 50% 1RM) repetitions to failure seem to be essential for increases in muscle strength and mass. The current notion is that there are no absolute beneficial effects on hypertrophy when performing resistance exercise training throughout a full ROM compared to partial ROM. Nonetheless, from a functional perspective full ROM should be addressed if possible.

## Author Contributions

CV: original idea. CV and DA: development and formulation of concept, draft, and critical revision. Both authors contributed to the article and approved the submitted version.

## Conflict of Interest

DA was employed by company Kieser Training AG. The remaining author declares that the research was conducted in the absence of any commercial or financial relationships that could be construed as a potential conflict of interest.

## Publisher's Note

All claims expressed in this article are solely those of the authors and do not necessarily represent those of their affiliated organizations, or those of the publisher, the editors and the reviewers. Any product that may be evaluated in this article, or claim that may be made by its manufacturer, is not guaranteed or endorsed by the publisher.
